# Down-regulation of neuregulin2 (NRG2) following spinal cord injury in C57BL/6 mice: Its implications in therapeutic potential

**DOI:** 10.22038/ijbms.2025.83787.18131

**Published:** 2025

**Authors:** Kai-Ye Hua, Yan-Jun Liu, Qi Ke, Quan Song, Shuai Zhang, Wei-Jiang Zhao

**Affiliations:** 1 Wuxi School of Medicine, Jiangnan University, Wuxi 214122, Jiangsu, P.R. China; 2 Wuxi No.2 People’s Hospital (Jiangnan University Medical Center), Wuxi 214002, Jiangsu, P.R. China; 3 Center for Neuroscience, Shantou University Medical College, Shantou 515041, Guangdong, P.R. China

**Keywords:** C57BL/6 mouse, Migration, Nerve regeneration, NRG2, PI3K-Akt, Spinal cord injury (SCI)

## Abstract

**Objective(s):**

This study aims to elucidate the alterations in neuregulin 2 (NRG2) and its receptor ErbB4 following spinal cord injury (SCI), as well as to investigate the neuroprotective mechanisms of NRG2 in neurons.

**Materials and Methods:**

Dataset GSE93561 was analyzed to verify the changes of NRG2-ErbB4 signaling pathway in mice following SCI. The levels of Iba-1 and NRG2 were analyzed by immunohistochemistry, and NRG2 and pErbB4 protein levels were detected by western blot. HT22 cells were scratched and treated with NRG2 dosed from 0–5 nM. Cell mobility was measured at the time point of 0, 24, and 48 hr after scratch. Additionally, western blot was used to detect the protein levels of pErbB4 and pAkt1 at 48 hr.

**Results:**

By analyzing dataset GSE93561, NRG1, NRG2, and NRG3 were found to be decreased to different degrees post-SCI in mice. The results of immunohistochemistry showed that the level of Iba-1 in the injured core area was significantly increased 8 weeks post-SCI. Western blot analysis showed that the protein levels of NRG2 and pErbB4 were decreased significantly post-SCI. NRG2 promoted HT22 cell migration and dose-dependently increased pErbB4 and pAkt1 protein levels at doses ranging from 0-5 nM.

**Conclusion:**

The NRG2-ErbB4 signaling pathway was inhibited after SCI in mice. NRG2 promotes the healing of HT22 cells after scratch injury, and the mechanism of NRG2 in the treatment of SCI may be ascribed to the activation of PI3K-Akt signaling pathways downstream to NRG2.

## Introduction

Spinal cord injury (SCI) represents a significant traumatic pathology of the nervous system, characterized by the disruption of the spinal cord’s microstructure. This disruption leads to restricted neuronal regeneration and inadequate functional recovery in adult mammals (1, 2). The degree and severity of sensory, motor, and autonomic function loss resulting from SCI are contingent not only upon the extent of the injury but also on its completeness (3). Presently, there is no definitive treatment for SCI. However, therapeutic strategies involving cellular and molecular approaches, as well as rehabilitation training, are under development, with several undergoing clinical trials (4). Findings have confirmed that exosomes derived from neural stem cells undergoing necroptosis play a crucial role in cellular communication following SCI. Specifically, when there is an up-regulation of Tuberous sclerosis 2 (TSC2) in the recipient cells, it contributes to functional recovery of SCI (5). 

Apoptosis following SCI has been demonstrated to be intricately associated with microglia and degenerating oligodendrocytes (6, 7). Microglia are integral to the pathophysiological processes occurring post-SCI. Initially, microglia rapidly respond to SCI by engaging in phagocytosis to eliminate invading pathogens and cellular debris, thereby contributing to neuroprotection and repair. However, persistent activation of microglia can have detrimental effects on neuronal cells by facilitating the aggregation and infiltration of inflammatory cells, which subsequently exacerbate the inflammatory response and mediate secondary injury (8).

Neuregulins (NRGs), members of the epidermal growth factor (EGF) superfamily, are involved in intercellular signaling across various systems, including the nervous system, heart, and breast (9). The ErbB tyrosine kinase receptor family, comprising four receptor subtypes (ErbB1-4), serves as the endogenous receptor for NRGs. The NRG family consists of four identified members: NRG1, NRG2, NRG3, and NRG4 (10). NRG1, a prominent member of the neuregulin family, is predominantly secreted by neurons and glial cells. It plays a crucial role in nerve generation and differentiation, as well as in the regulation of synaptic and neuromuscular connections (11). The NRG1/ErbB signaling pathway is instrumental in modulating Schwann cell proliferation, migration, and myelination (12). Following SCI, ErbB receptor signaling is pivotal in facilitating the conversion of oligodendrocyte precursor cells into Schwann cells (13). NRG1 fosters an optimal environment for SCI repair by diminishing antibody deposition and the expression of pro-inflammatory cytokines and chemokines while concurrently up-regulating pro-regenerative mediators (14).

NRG2, another significant member of the NRG family, shares structural similarities with NRG1, and its expression in the adult rodent brain complements that of NRG1 (15). In the adult brain, NRG2 is sustainably expressed in three regions, namely the cerebellum, olfactory bulb, and dentate gyrus of the hippocampus (15). Unlike NRG1, which is expressed in both axons and dendrites, NRG2 is predominantly localized in neuronal dendrites (16). Similar to NRG1, NRG2 activates ErbB1 and ErbB2 through direct binding to ErbB3 or ErbB4, forming heterodimers with each receptor individually (17). NRG2, secreted by astrocytes, can bind to ErbB3 on neurons, thereby promoting neuronal survival and neurite extension *in vitro* (18). Furthermore, NRG2 influences the migratory behavior of glioma and glioblastoma cells, significantly enhancing their motility (19). NRG2 is highly expressed in glioma tissues across various grades and modulates the expression of GFAP in glioma cells via the Akt signaling pathway, impacting the prognosis of glioma patients (20). In NRG2 knockout mice, there are notable dopamine imbalances in the dorsal striatum and medial prefrontal cortex, altered glutamate transmission in the hippocampus, numerous behavioral abnormalities, and a significant response to clozapine treatment, indicating NRG2’s critical role in synaptic transmission (21).

While the role of NRG1 in neurophysiology, behavior, and the treatment of nervous system disorders has been extensively investigated, there is a relative paucity of research on the related functions of NRG2. Compared to NRG1, NRG2 possesses a smaller molecular weight and exhibits more specific receptor binding, leading us to hypothesize that NRG2 may be more efficacious in the treatment of SCI. In this study, we analyzed the dataset GSE93561 to assess the attenuation of the NRG2-ErbB4 signaling pathway following SCI in mice. Immunohistochemistry was employed to examine the effects of SCI on NRG2 expression and the inflammatory response in mice. Subsequently, recombinant NRG2 was administered following cellular injury to investigate the impact of varying concentrations of NRG2 on the motility of HT22 neurons. Additionally, western blot analysis was utilized to explore the relevant pathways and mechanisms underlying NRG2’s neuroprotective effects. This research may provide valuable insights for the treatment of SCI via the NRG2-ErbB4 signaling pathway. 

## Materials and Methods

### Materials

HT-22 mouse hippocampal neuronal cell lines (Cat# CL-0595) were purchased from Procell, Wuhan, China. Female C57BL/6 mice were purchased from Guangdong Medical Laboratory Animal Center (Guangzhou, China) and maintained in the Animal Center of Shantou University Medical College (SUMC). The Institutional Animal Care and Use Committee of SUMC approved the animal experimental protocols (No. SUMC2014-004). Penicillin/streptomycin mixture (Cat# C022) was purchased from Beyotime, Shanghai, China. Fetal bovine serum (FBS, Cat# 11011-8611) was purchased from Tianhang Biological Technology Company Ltd., Zhejiang, China. The Dulbecco’s modified Eagle’s medium (DMEM) (Cat# D211113) for cell culture was purchased from BasalMedia, Shanghai, China. Recombinant NRG2β (Cat# NRG2-363H Human) was purchased from Creative BioMart, Shirley, NY, USA. AEC enzyme substrate kit (Cat# ZLI-9036) and Mouse/Rabbit Enhanced Polymer assay system (Cat# PV-9000) were purchased from ZSGB-BIO, Beijing, China.

### GEO dataset analysis

The GSE93561 dataset was downloaded from the GEO database to analyze the differences in the expression levels of transcriptome NRG1, NRG2, and NRG3 and their receptor ErbB4 in young and old mice subjected to SCI.

### Preparation of mouse spinal cord injury model

Three-month-old wild-type C57BL/6 female mice were randomly divided into two groups, denoted as the sham (Sham) group and spinal cord injury (SCI) group. All SCI surgeries were performed under aseptic conditions as described (22, 23). Briefly, mice were anesthetized by intraperitoneal injection of a mixture of ketamine (100 mg/kg, Fujian Gutian Pharmaceutical, Ningde, Fujian, China) and xylazine (5 mg/kg, Sigma-Aldrich). The dorsal skin was shaved and disinfected, followed by exposure of the dorsal aspect of the spinal column. Laminectomy was performed at the T9-T11 levels to expose the spinal segment while ensuring the dura mater remained intact. The spinal cord was severely compressed for five seconds, using forceps (RWD Life Science Co., Ltd, Shenzhen, China) under standardized and controlled conditions. Successful modeling was indicated by the occurrence of spasmodic tail wagging, limb paralysis post-retraction, and body flapping at the moment of injury. In the sham group, the spinal cord was exposed without compromising the dura mater. The mice were housed in cages, and their bladders were manually emptied once daily until reflexive bladder function was restored. Eight weeks post-injury, mice were utilized for western blot and histochemical analyses. For western blot analysis, spinal cord tissues were dissected and stored at -80 °C. For morphological assessment, mice underwent transcardial perfusion with a 4% paraformaldehyde solution in phosphate-buffered saline (PBS), and the spinal cord tissues were subsequently immersed in a 30% sucrose solution until they sank (24).

### Hematoxylin-eosin (HE) and Immunohistochemistry staining

Frozen sections, each 8 μm thick, were placed in an oven at 55 °C for 12 hr and subsequently allowed to cool to room temperature. HE staining was undertaken using the HE staining kit (Solarbio, G1121). For immunohistochemistry staining, the sections were then washed in a PBS solution (Cat# P1010, Solarbio, Beijing, China) for 15 min. Following this, they were immersed in an antigen retrieval solution (Cat# C1010, Solarbio, Beijing, China) that had been preheated in a 99 °C water bath for 15 min, and the antigen retrieval process was conducted for 40 min. The sections were then cooled to room temperature. To inhibit endogenous peroxidase activity, a 3% H_2_O_2_ solution was applied, and non-specific binding was blocked using 10% bovine serum (Cat# I0910, Solarbio, Beijing, China) for 20 min. The sections were incubated overnight at 4 °C with Anti-Iba-1 (1:100; Cat# PB0517, Boster Biotech, Wuhan, China) and anti-NRG2 (1:50; Cat# SC-398594, Santa Cruz Biotech, Santa Cruz, CA, USA) antibodies, respectively. A reaction enhancer (Cat# PV-9000, ZSGB-BIO, Beijing, China) was applied, followed by visualization of the antigen-antibody complexes using an AEC kit (Cat# ZLI-9036, Solarbio, Beijing, China). Images were captured using an optical microscope (DN-10B, Jiangnan, Jiangsu, China) (20).

### Culture of HT22 cells

Cells were maintained in DMEM supplemented with 50 U/ml penicillin/streptomycin mixture (Beijing Solarbio Science and Technology Co., Ltd.) and 10% FBS. The cells were routinely cultured in 75 cm^2^ cell culture plates (Corning Inc.) at 37 °C in a humidified incubator (HERA cell 240, Thermo Scientific, Germany) with 5% CO_2_.

### Wound healing assay

HT22 cells were routinely cultured until reaching approximately 90% confluence. Subsequently, the cells were treated with 0.25% trypsin-EDTA (Solarbio) for enzymatic digestion. The digested cells were then centrifuged in a serum-containing medium at 900 rpm for three minutes. Following centrifugation, the supernatant was discarded, and the cell pellet was resuspended in fresh serum-containing medium. The cells were seeded into a 48-well plate at a density of 1 × 10^5^ cells per well. A scratch was made in the center of each well using a sterile 10 μl micropipette tip. The scratched area was washed with serum-free DMEM medium to remove detached cells. Subsequently, 300 μl of serum-free DMEM medium containing 0, 2, 5, and 10 nM NRG2 protein was added to the cells according to their respective groups, with each concentration tested in triplicate. Wound healing was monitored at 0, 24, and 48 hr using an inverted microscope (BX51, Olympus, Japan). The migration rate was calculated using the formula: healing rate = [(initial scratch area – final scratch area) / initial scratch area] × 100%. ImageJ software (NIH *Image J *system, Bethesda, MD, Iran) was utilized to analyze the scratch healing rate (19).

### Western blot

After 48 hr of conducting the scratch test, cells in each well were photographed and subsequently lysed in a 50 μl solution comprising a RIPA buffer (Cat# R0010), protease phosphate inhibitor (Cat# P1260), and PMSF (Cat# IP0280) at a ratio of 100:1:1. All reagents were procured from Solarbio, Beijing, China. The lysate was centrifuged at 14,000 *g* for 15 min at 4 ℃. The resulting supernatant was collected for protein quantification using the BCA assay. The protein sample was then denatured in a metal bath at 100 ℃ for ten minutes. Subsequently, 20 μg of protein was loaded into each well for 10% SDS-PAGE electrophoresis, followed by electroblotting onto a polyvinylidene difluoride (PVDF) membrane (Millipore, MA, USA). The PVDF membrane was blocked at room temperature with a blocking solution for one hour. Thereafter, mouse anti-pAkt1 (1:500; Cat# SC-293125, Santa Cruz Biotech), NRG2 (1:500; Cat# SC-398594, Santa Cruz Biotech), GAPDH (1:500; Cat# SC-33763, Santa Cruz Biotech), β-actin (1:500; Cat# SC-47778, Santa Cruz Biotech), and rabbit anti-pErbB4 antibodies (1:500; Cat# AP0034, ABclonal Biotechnology Co., Ltd., Wuhan, China) were added and incubated overnight at 4 ℃. Goat anti-rabbit (1:1000; Cat# bs-40295G, Bioss Biotechnology, Beijing, China) and goat anti-mouse (1:1000; Cat# bs-40296G, Bioss Biotechnology) secondary antibodies were incubated at room temperature for one hour, followed by ECL detection.

### Statistical analysis

Statistical analyses were conducted utilizing GraphPad Prism 9.0 software (GraphPad Software, Inc.). To evaluate differences between two groups, a two-tailed Student’s *t*-test was employed, while differences among multiple groups were assessed using a two-way analysis of variance (ANOVA). *P*<0.05 was considered indicative of statistical significance for all tests performed. 

## Results

### Expression of NRG1, NRG2, NRG3 and their receptor ErbB4 in mice after spinal cord injury

**Figure 1:**
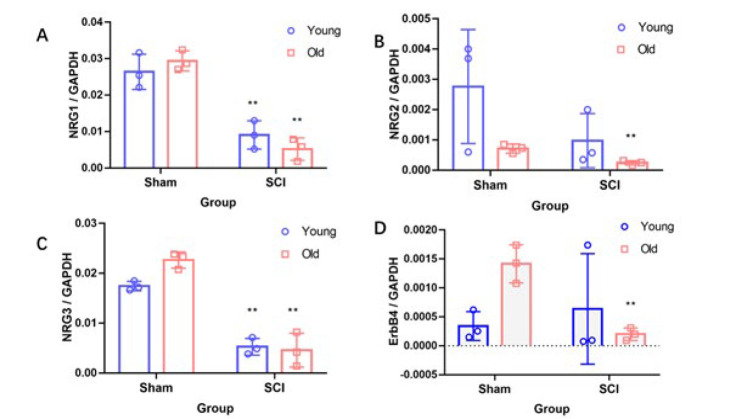
GEO-based analysis of the expression levels of NRG1-3 and ErbB4 in spinal cord injury (SCI)

To investigate alterations in the protein levels of the NRG family and ErbB4 following SCI, we accessed dataset GSE93561 from the GEO database. Our analysis revealed that, compared to the sham operation group, the levels of NRG1 and NRG3 proteins were significantly reduced in both young and elderly mice post-SCI (*P*<0.01) (Figure 1A, C). In contrast, NRG2 exhibited a significant decrease only in elderly mice following SCI (*P*<0.01), with no notable difference observed in young mice (*P*>0.05) (Figure 1B). Additionally, the expression of ErbB4 protein in elderly mice post-SCI was significantly lower than that in the sham operation group (*P*<0.01) (Figure 1D). These findings suggest that NRG2 may have a critical role in SCI, particularly in elderly mice (*P*<0.01) (Figure 1).

### Morphological alterations and Iba-1 expression following SCI

**Figure 2:**
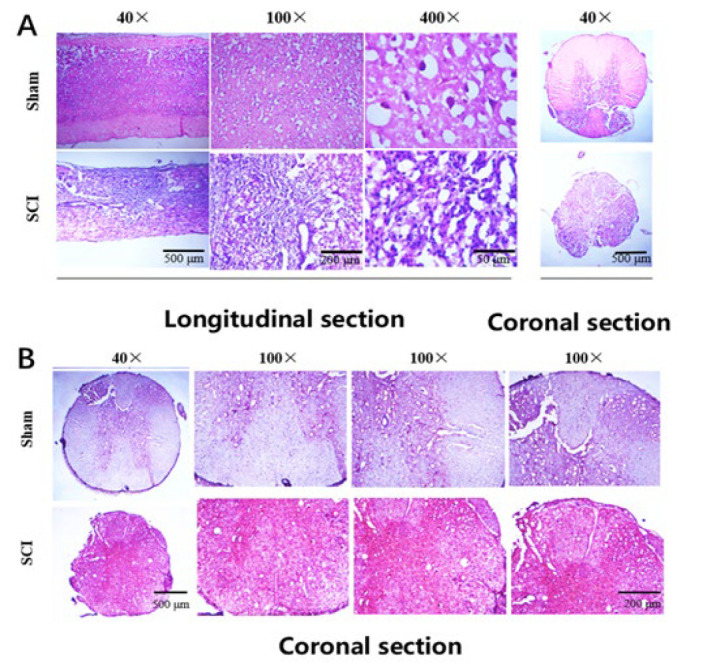
Morphological changes and microglia activation in the injured core area post-SCI

HE staining was employed to observe pathological changes at eight weeks post-SCI. In comparison to the sham-operated group, the SCI group exhibited blurred boundaries between gray and white matter, nuclear atrophy in certain regions, disordered or disrupted nerve fiber arrangement, and the presence of cavities and cystic changes in the injured core area (Figure 2A). Immunohistochemical analysis revealed a uniformly strong positive expression of the microglial marker Iba-1 in the SCI group (Figure 2B), indicating extensive microglial activation following SCI. 

### Expressions of NRG2 and ErbB4 in vivo

**Figure 3:**
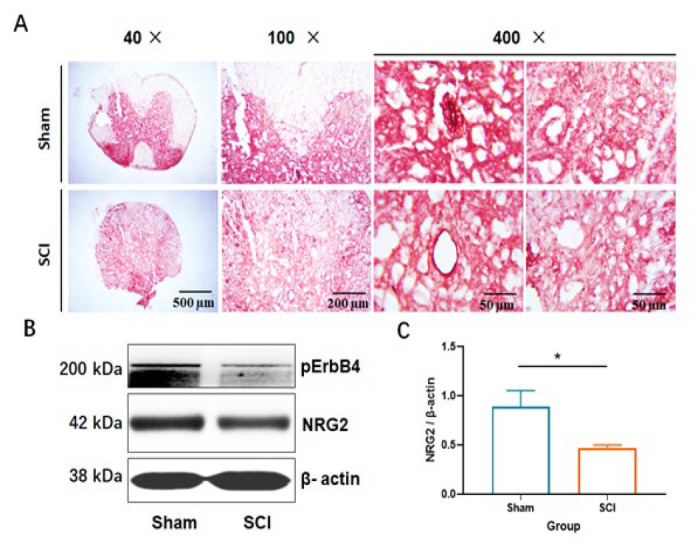
Impact of SCI on the expression of NRG2 and associated signaling molecules at the spinal cord epicenter

*In vivo* immunohistochemical analysis of NRG2 expression revealed that SCI led to a marked decrease in NRG2 expression at the epicenter of the spinal cord (Figure 3A). To substantiate these findings, proteins were extracted from spinal cord tissues of both the sham and SCI groups, and the expression levels of NRG2 and phosphorylated ErbB4 (pErbB4) were assessed via western blot analysis. Relative to the sham group, the expression levels of NRG2 and pErbB4 were diminished following SCI (Figure 3B). Notably, NRG2 levels in the SCI group were significantly lower than those in the sham group (Figure 3C). These results collectively suggest a disruption of the NRG2-ErbB4 signaling pathway subsequent to SCI.

### Effect of NRG2 on wound healing of HT22 cells

**Figure 4:**
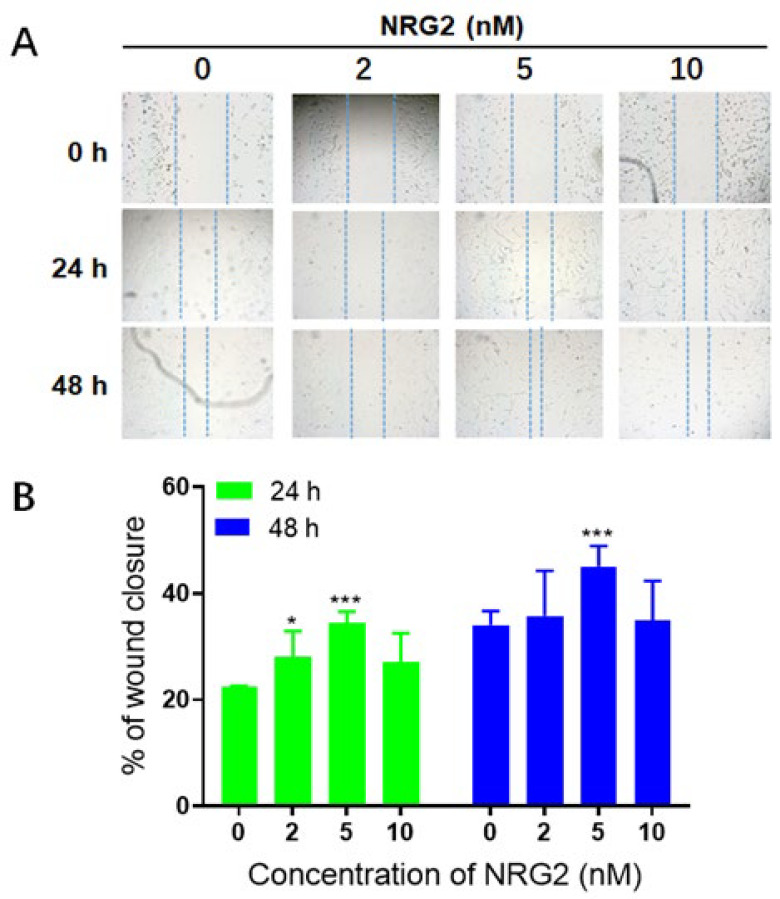
Analysis of the migration rate of HT22 cells following the administration of varying concentrations of NRG2

Apoptosis, or the loss of neuronal cells, is a significant contributor to the exacerbation and irreversibility of neuronal injury following SCI. In this study, we employed cell scratch assays to simulate the process of SCI and observed that there was no detectable difference in gap width immediately (0 hr) after the scratch. However, after 24 and 48 hr of culture, NRG2 at concentrations of 2, 5, and 10 nM facilitated gap closure in HT22 hippocampal neurons. Notably, NRG2 enhanced the wound healing capacity of HT22 hippocampal neurons in a dose-dependent manner when compared to the control group at concentrations ranging from 0 to 5 nM, with the most pronounced effect observed at 5 nM at both time points tested (Figure 4B, *P*<0.001). The results from the cell scratch assay indicated that exogenous NRG2 administration significantly promoted the migration of HT22 cells, thereby accelerating the healing rate in this experimental model of SCI. Within the first 24 hr post-administration, drug concentrations between 0 and 5 nM demonstrated a positive correlation with healing efficacy. However, the degree of promotion diminished, and the effect was attenuated at concentrations exceeding 10 nM. 

### Effect of NRG2 on the phosphorylation-dependent activation of ErbB4 and Akt1 post-cell scratch

**Figure 5:**
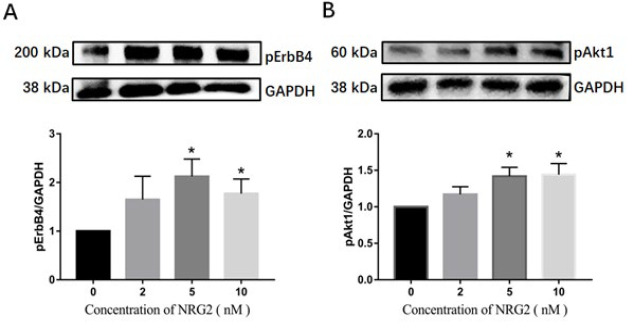
Western blot analysis of the effect of NRG2 on pErbB4 and pAkt1 post scratch of HT22 cells

Western blot analysis revealed a significant increase in pErbB4 levels in the 5 nM and 10 nM NRG2 treatment groups 48 hr post-administration compared to the solvent control group (Figure 5A) (*P*<0.05). These findings suggest that NRG2 exerts an activating effect on the ErbB4 receptor. Furthermore, western blot was employed to assess changes in the levels of activated Akt1 (pAkt1) protein, exploring the impact of NRG2 on its downstream signaling pathway. The results indicated a significant elevation in pAkt levels in HT22 cells treated with 5 nM and 10 nM NRG2 for 48 hr, relative to the solvent control group (Figure 5B) (*P*<0.05). These findings demonstrate that NRG2 interacts with its receptor ErbB4 to activate the PI3K-AKT signaling pathway post-cell scratch.

## Discussion

Clinically, SCI can result in cognitive dysfunction and significantly impact both the quality of life and survival duration of affected patients (25). Research has demonstrated that NRG1 facilitates the repair of SCI by up-regulating the PI3K-AKT-mTOR pathway, thereby converting reactive astrocytes into oligodendrocyte lineage cells (26). Recent studies have suggested that neuregulin-2 (NRG2) also plays a crucial role in neuronal regeneration and repair following SCI (27). This study utilized a GEO database to investigate the expression of NRG1, NRG2, NRG3, and their receptor ErbB4 in mice with SCI. Additionally, our *in vivo* findings confirmed the down-regulation of NRG2 and a reduced level of pErbB4 in mice subjected to SCI. On this basis, HT22 hippocampal neurons were subjected to scratch assays to simulate *in vitro* SCI, assessing the therapeutic potential of NRG2. It was confirmed that NRG2 enhances the mobility of HT22 neurons and promotes neuronal migration in a dose-dependent manner at concentrations ranging from 0 to 5 nM.

Previous research has demonstrated that long-term SCI leads to significant activation of microglia, accompanied by disruptions in NRG1 signaling pathways (24). *In vivo* studies have further corroborated that the population of microglial cells in SCI-afflicted mice is markedly increased. Relevant studies suggest that excessive microglial activation influences the aggregation of macrophages and T lymphocytes, exacerbates the inflammatory response within the nervous system, accelerates neuronal death, and impedes nerve regeneration and repair (25). There is a notable decrease in the expression of NRG2 and its receptor, prompting speculation that NRG2 may play a crucial role in neuronal regeneration post-SCI by activating its receptor, ErbB4.

Compared with NRG1, NRG2 possesses a smaller molecular weight and exhibits more specific receptor binding. NRG2 can bind to the neuronal ErbB3 receptor, thereby promoting neuronal survival and axon extension *in vitro* (28). 

## Conclusion

We previously demonstrated that NRG2 is highly expressed in grades I-III gliomas and facilitates the migration of human glioma/glioblastoma cell lines, including SHG44, U251, and U-87 MG (19). Furthermore, NRG2 has been shown to facilitate neuronal cell migration, thereby contributing to neuronal repair (29). Concurrently, the activation of ErbB4 inhibits the NF-κB pathway, leading to a reduction in neuroinflammation through the down-regulation of microglial polarization (11). Previous studies have demonstrated that NRG2 is involved in enhancing neuronal survival and neurite outgrowth (18). Based on these findings, *in vitro* experiments conducted using mouse hippocampal HT22 cells revealed that NRG2 interaction with its receptor ErbB4 activates the PI3K-AKT signaling pathway, contributing to neuronal migration. These findings collectively indicate that NRG2 signaling is crucial in mitigating neuroinflammation and other neuropathological processes associated with SCI. 

Modulation of the relevant signaling pathways by exogenous NRG2 may enhance neuronal migration and regeneration, thereby facilitating the restoration of neural function following SCI. Additionally, the involvement of its receptor ErbB4 and the downstream signaling molecule Akt1 is significant in these processes. Recent reports indicate that the targeted activation of the ErbB4 receptor using a small molecule alleviates D-galactose-induced neuronal senescence both *in vitro* and *in vivo *(30). Consequently, small molecules that mimic NRG2, identified through virtual screening, may be employed via intravenous or localized administration for the future treatment of SCI (31).

The presence of similar results in cell lines other than HT22 cells remains unverified. Employing PI3K or AKT inhibitors could bolster the assertion concerning the specificity of NRG2’s effects. While it has been established that NRG2 plays a significant role in SCI, particularly in elderly mice, further investigation is required in young mice to substantiate this theory. Additionally, the impact of NRG2 on SCI treatment, especially regarding behavioral improvement, necessitates further validation through animal model experiments.

The gene expression levels of NRG1 (A), NRG2 (B), NRG3 (C), and ErbB4 (D) were assessed in both young and old mice with SCI. Data are expressed as means ± standard deviation (SD) from three independent experiments, with statistical significance indicated by * *P*<0.05 and ** *P*<0.01 compared to the control group. NRG2: Neuregulin 2

(A) Hematoxylin-eosin (HE) staining in the spinal cord of mice subjected to either sham operation or SCI. (B) Immunohistochemical staining of Iba-1 in the spinal cord of mice subjected to either sham operation or SCI.

(A) Immunohistochemical staining of NRG2 in the spinal cord of mice subjected to either a sham operation or SCI. (B) Western blot analysis of pErbB4 and NRG2 in the spinal cord of mice subjected to either sham operation or SCI. (C) Statistical analysis of NRG2 in the spinal cords of mice subjected to either sham operation or SCI. Data are presented as means ± SEM from three independent experiments (* *P*<0.05). NRG2: Neuregulin 2

(A) Optical microscope images depict wound closure after a cell scratch assay in response to NRG2 treatment in HT22 cells, observed at a magnification of ×100. (B) Data are expressed as means ± SEM from five independent experiments, with statistical significance denoted as follows: * *P*<0.05 and *** *P*<0.001 compared to the control group.

NRG2: Neuregulin 2

Protein expression level of pErbB4 (A) and pAkt1 (B) in scratched HT22 cells treated with 0,2,5,10 nM NRG2 for 48 hr. (A) Data are presented as means ± SEM from five independent experiments; (B) Data are presented as means ± SEM from four independent experiments. * *P*<0.05 vs control group. NRG2: Neuregulin 2

## Declaration

We have not used any AI tools or technologies to prepare this manuscript.
